# Upper limb pediatric fractures in 22 tertiary children's hospitals, China: a multicenter epidemiological investigation and economic factor analysis of 32,832 hospitalized children

**DOI:** 10.1186/s13018-022-03159-5

**Published:** 2022-06-03

**Authors:** Xin Qiu, Hansheng Deng, Zhenhui Zhao, Shuaidan Zeng, Yueping Zeng, Xinyu Wang, Hui Xu, Weiqing Li, Xiaodi Chen, Qisong Yang, Jiaxin Zhao, Shicheng Li, Zhiwen Cui, Yu Tang, Shuting Cui, Min Liu, Yiyuan Sun, Guoshuang Feng, Gen Tang, Zhu Xiong, Shengping Tang, Futang Research Center of Pediatric Development (FRCPD)

**Affiliations:** 1 Department of Pediatric Orthopedics, Shenzhen Children’s Hospital of China Medical University, Shenzhen, Guangdong Province People’s Republic of China; 2 Big Data Center, Beijing Children’s Hospital, Capital Medical University, National Center for Children’s Health, Beijing, People’s Republic of China; 3 Hefei Cancer Hospital, Chinese Academy of Science, Hefei, People’s Republic of China; 4 Guangxi Medical University, Nanning, Guangxi Province People’s Republic of China

**Keywords:** Fractures, Epidemiological investigation, Economic factor

## Abstract

**Background:**

Fractures are the most common type of unintentional injury in children, with traumatic upper limb fractures accounting for approximately 80% of all childhood fractures. Many epidemiological investigations of upper limb fractures in children have been conducted, but with the development of society, the patterns of childhood fractures may have changed. This study aimed to analyze the epidemiology and economic cost factors of upper limb fractures in Chinese children.

**Methods:**

We retrospectively reviewed children with upper limb fractures or old upper limb fractures hospitalized between December 1, 2015, and December 31, 2019, in 22 tertiary children’s hospitals, under China’s Futang Research Center of Pediatric Development. We used the ICD10 codes on the front sheet of their medical records to identify cases and extracted data on age, sex, injury cause, fracture site, treatment, the year of admission and discharge, visiting time, and various costs during hospitalization from the medical record.

**Results:**

A total of 32,439 children (21,478 boys and 10,961 girls) were identified, of whom 32,080 had fresh fractures and 359 had old fractures. The peak age was 3–6 years in both sexes. A total of 4788 were infants, 14,320 were preschoolers, 10,499 were in of primary school age, and 2832 were adolescent. Fractures were most frequent in autumn (August to October). Admissions peaked at 0 o’clock. Among the 32,080 children with fresh upper limb fractures, the most common fracture site was the distal humerus, with a total of 20,090 fracture events including 13,134 humeral supracondylar fractures and 4914 lateral humeral condyle fractures. The most common cause of injuries was falling over. The most common joint dislocation accompanying upper limb fractures occurred in the elbow, involving 254 cases. Surgery was performed in 31,274 children, and 806 did not receive surgery. Among those with clear operative records, 10,962 children were treated with open reduction and 18,066 with closed reduction. The number of cases was largest in the East China region (Anhui Province, Shandong Province, Jiangsu Province, Zhejiang Province, and Fujian Province), with 12,065 cases overall. Among the 359 children with old fractures, 118 were admitted with a diagnosis of “old humerus fracture,” accounting for the highest proportion; 244 underwent surgical open reduction, 16.16% of whom had osteotomy. For the children with fresh fractures, the average total hospital cost was 10,994 yuan, and the highest average total hospital cost was 14,053 yuan, for humeral shaft fractures. For the children with old fractures, the average total hospital cost was 15,151 yuan, and the highest average total hospital cost was 20,698 yuan, for old ulna fractures. Cost of materials was the principle factor affecting total hospital cost, followed by surgery and anesthesia costs, both in children with fresh fractures and those with old fractures. Significant differences were observed in all hospital costs (*P* < 0.001) except treatment costs (*P* = 0.702), between children with fresh fractures and those with old fractures. Among the 32,439 children, full self-payment accounted for the highest proportion of all payment methods, involving 17,088 cases, with an average cost of 11,111 yuan.

**Conclusion:**

Information on the epidemiological characteristics of childhood fractures suggests that health and safety education and protective measures should be strengthened to prevent upper limb fractures in children. For both fresh and old fractures, the cost of materials was the principal factor affecting total hospital cost, followed by surgery and anesthesia costs. The overall average total hospital cost is higher in children with old fractures than in children with fresh fractures. Among all children, full self-payment, at 53% of children, accounted for the highest proportion of all payment methods. Hospital costs are a headache for those families who will pay on their own. It can lead to a delayed treatment and unhealed fractures or malunion in some children. Therefore, the child trauma care system and training on fractures need to be improved, to reduce the late presentation of fractures. These combined measures will improve children’s quality of life, reduce the expenditure of families, and decrease the public health burden. To provide better medical services for children, authorities must improve the allocation of health resources, establish a comprehensive medical security system for children, and set up more child trauma centers.

## Introduction

Childhood unintentional injuries are a major global public health issue, with numerous children and adolescents around the world dying from accidental injuries and economic loss of between 500,000 and 9.5 million US dollars per year [1]. Fractures are the most common type of unintentional injury in children, accounting for 10% to 25% of all injuries [2], and the incidence of childhood fractures has increased over time [3]. Childhood fracture types vary, depending on local climate, culture, and casual activities, and the causes of fractures also vary between countries or even across different regions of the same country [4, 5]. Traumatic upper limb fractures represent approximately 80% of all fractures in children [6, 7]. China has the second largest child population in the world, which makes up 12.9% of the global child population [8]. However, few studies have been conducted on overall fracture site distribution and epidemic trends in Chinese children and adolescents, and large-sample studies and epidemiological data on old upper limb fractures in children are especially lacking. Economic cost analysis of children’s admissions due to upper limb fractures is also lacking. We aimed to present the epidemiological data of hospitalized children with upper limb fractures in China and provide scientific preventive strategies for reducing the incidence of fresh and old upper limb fractures in children. In addition, we investigated the economic burden of upper limb fractures in children and its influencing factors, to provide a scientific basis for improving children’s quality of life and formulating health policy to reduce the economic burden of upper limb fractures.

## Methods

We retrospectively reviewed children who had presented with recent or old upper limb fractures and were hospitalized between December 1, 2015, and December 31, 2019, in any of the 22 tertiary children’s hospitals under China’s Futang Research Center of Pediatric Development [9]. We used the front sheet of their medical records to find cases according to ICD code, and extracted data from the medical files on age, sex, cause of injury, fracture site, treatment, year of admission and discharge, visiting time, and various costs during hospitalization.

The children were classified into the following groups by age: infants (< 2 years), preschool children (2–5 years), school children (6–11 years), and adolescents (12–18 years). Based on mechanism of injury, the causes of fractures were categorized as follows: falling over, falling from a height, traffic accident, being struck or hit, other reasons, and unknown.

Fresh fractures included the following types by anatomical site: clavicle fracture; scapula fracture; proximal humerus fracture (including humeral surgical neck fracture, humeral anatomical neck fracture, humeral head fracture, proximal humeral epiphyseal separation fracture, and others); humeral shaft fracture; distal humerus fracture (including humeral supracondylar fracture, lateral humeral condyle fracture, lateral humeral epicondyle fracture, medial humeral epicondyle fracture, humeral intercondylar fracture, distal humeral epiphyseal separation fracture, humeral capitellum fracture, humeral trochlea fracture, and others); proximal ulna fracture (including ulnar olecranon fracture, ulnar coronoid process fracture, epiphyseal separation of the ulnar olecranon, and others); ulnar shaft fracture; distal ulna fracture (including ulnar styloid process fracture, distal ulnar epiphyseal separation fracture, and others); proximal radius fracture (including radial neck fracture, radial head fracture, and others); radial shaft fracture; distal radius fracture (including radial styloid process fracture, distal radial epiphyseal separation fracture, and others); and Monteggia fracture. In addition, we defined fresh fractures at the distal humerus, proximal ulna, and proximal radius as elbow fractures. We also recorded concomitant joint dislocations documented on the front sheet of the medical records.

Old fractures according to ICD code are: old humerus fracture, old radial or ulnar shaft fracture, old ulna fracture, old radius fracture, old Monteggia fracture, old lateral humeral condyle fracture, old humeral supracondylar fracture, old medial humeral condyle fracture.

We recorded the specific time and season (spring, summer, autumn, or winter) of admission of children with fresh or old fractures, and the geographic region (East China, South China, Central China, North China, Southwest China, Northeast China, or Northwest China) of children with fresh fractures.

For all included children, the following hospital costs were recorded: total hospital cost, nursing care cost, examination cost, treatment cost, surgery and anesthesia cost, medication cost, material costs, and other comprehensive care costs. Payment method was classified as self-payment, basic medical insurance for urban and rural residents, basic medical insurance for urban residents, basic medical insurance for urban employees, other social medical insurance, poverty assistance, full government payment, commercial medical insurance, and unknown.

## Results

### Age and sex

**Fig. 1:**
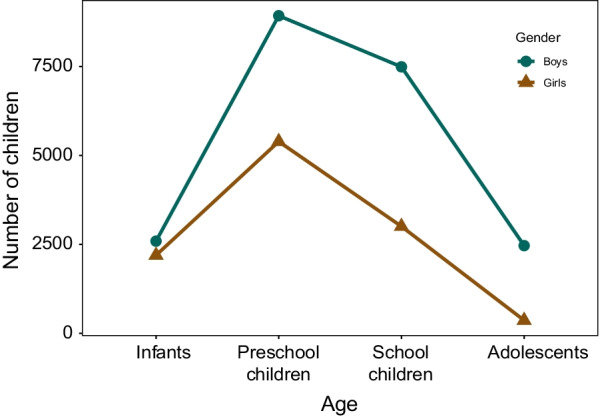
The proportion of male and female children by age group. This picture shows the proportion of male and female children in different age groups

A total of 32,439 children with fractures were included, with 21,478 boys and 10,961 girls. Boys were more than girls across all age groups. The number of children with fractures was highest in the preschool group and lowest in the adolescent group. In the infant group, there were 4788 fractures, with 2592 boys and 2196 girls. The number of cases increased with growth and development and peaked in preschoolers with 14,320 cases, with 8928 boys and 5392 girls. That figure decreased to 10,499 cases in the school child group, with 7492 boys and 3007 girls. Only 2832 cases were recorded in the adolescent group, with 2466 boys and 366 girls. Sex differences in the number of cases increased with age and were greatest in the adolescent group with a ratio of 1 girl to 6.7 boys (Fig. 1).

### Fracture site

**Fig. 2:**
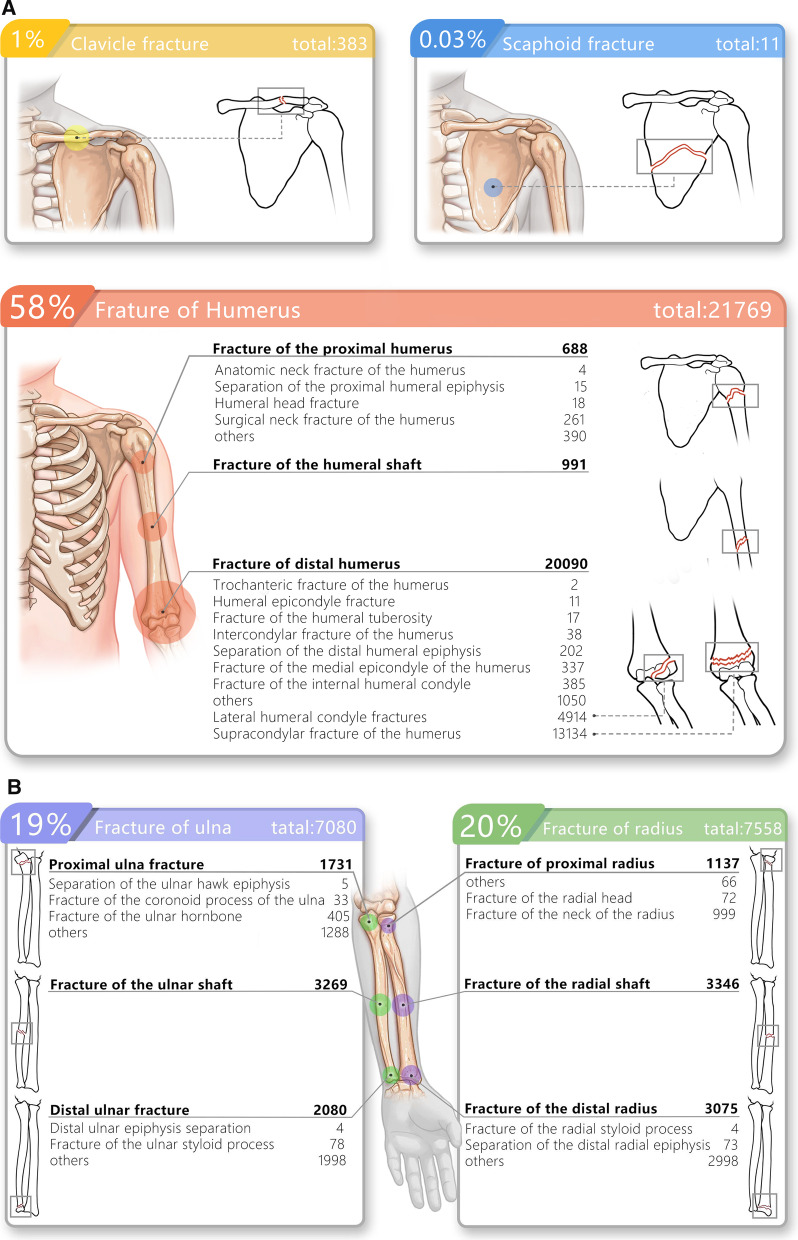

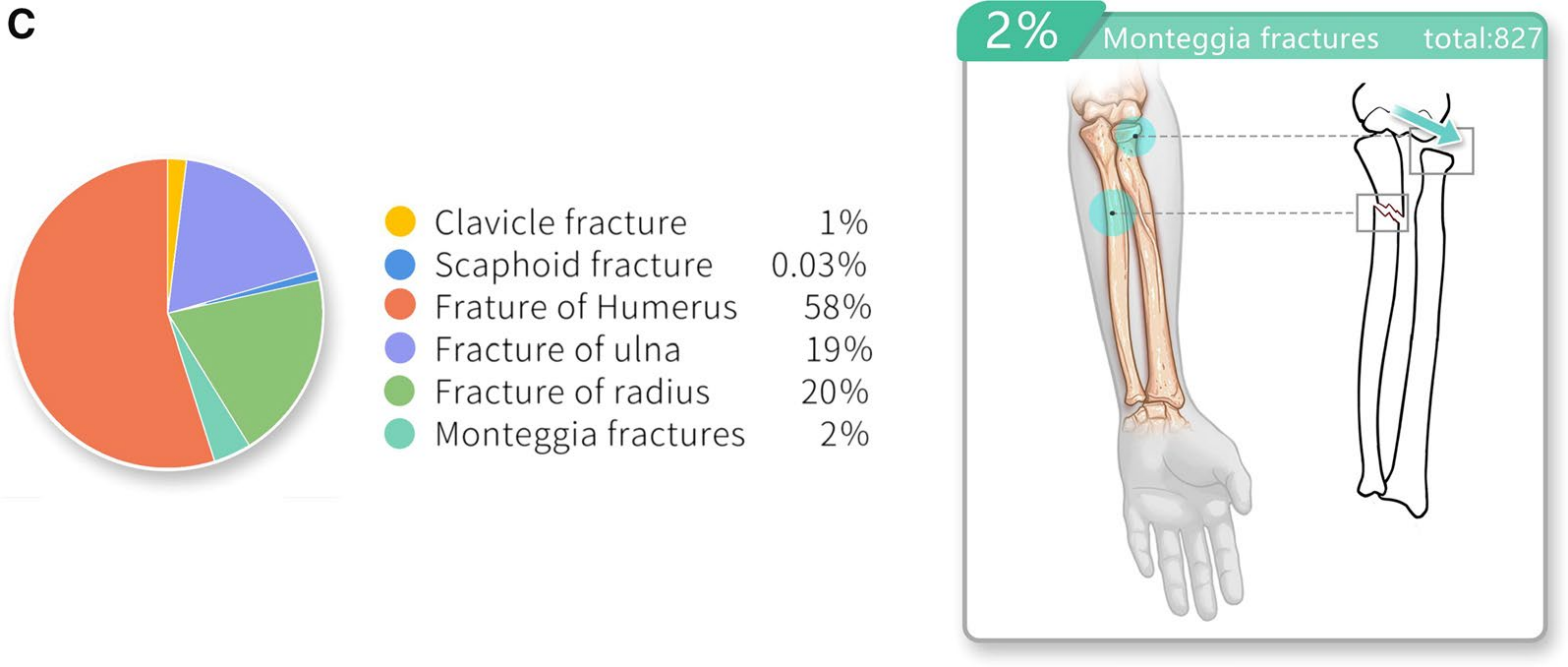
**A**–**C** The fracture sites of all patients. This picture shows the common fresh fracture sites in all patients

In the 32,080 children with fresh fractures, 37,627 fractures were recorded. The distal humerus was the most frequently affected site with 20,090 fractures in total, of which 13,134 were humeral supracondylar fractures, 4914 lateral humeral condyle fractures, and 1050 other distal humerus fractures. Moreover, the percentage of distal humerus fractures was highest in all four age groups. Radial shaft ranked second with 3346 fractures, followed by 3269 ulnar shaft fractures, 3075 distal radius fractures, 2080 distal ulna fractures, 1731 proximal ulna fractures, 1137 proximal radius fractures, 991 humeral shaft fractures, 827 Monteggia fractures, 688 proximal humerus fractures, 383 clavicle fractures, and 11 scapula fractures (Fig. 2A–C).

**Fig. 3:**
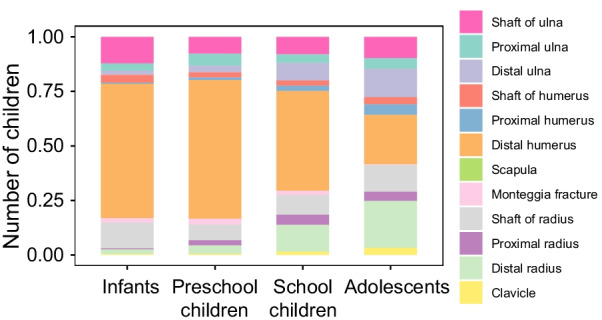
The epidemiology of traumatic fractures according to different age range groups. This picture illustrates the distribution characteristics of each fracture site in children of various age groups

Fresh distal humerus fractures were recorded in 12,783 boys and 7307 girls. These fractures were most commonly distributed in preschool children (10,181 fractures), followed by school children (5668 fractures), and were least in the adolescent group (832 fractures). Humeral supracondylar fractures accounted for the highest proportion of distal humerus fractures, involving 8070 boys and 5064 girls, and were most frequently distributed in preschool children (6538 fractures) and least in the adolescent group (356 fractures). Lateral humeral condyle fractures within the elbow joint affected 3354 boys and 1560 girls. They were most commonly distributed in preschool children (3098 fractures), followed by school children (1027 fractures), and were least in the adolescent group (57 fractures). There were 3346 radial shaft fractures involving 2415 boys and 931 girls, and these were most commonly distributed in preschool children (1146 fractures) and least in adolescents (445 fractures). Of the 3269 ulnar shaft fractures, 2322 were in boys and 947 in girls, and these were most commonly distributed in preschool children (1241 fractures) and least in adolescents (356 fractures) (Fig. 3).

The total of 22,958 fresh elbow fractures involved 14,509 boys and 8449 girls. These cases were most common in preschool children (11,438 cases), followed by school children (6730 cases), and least in adolescents (1174 cases).

**Fig. 4:**
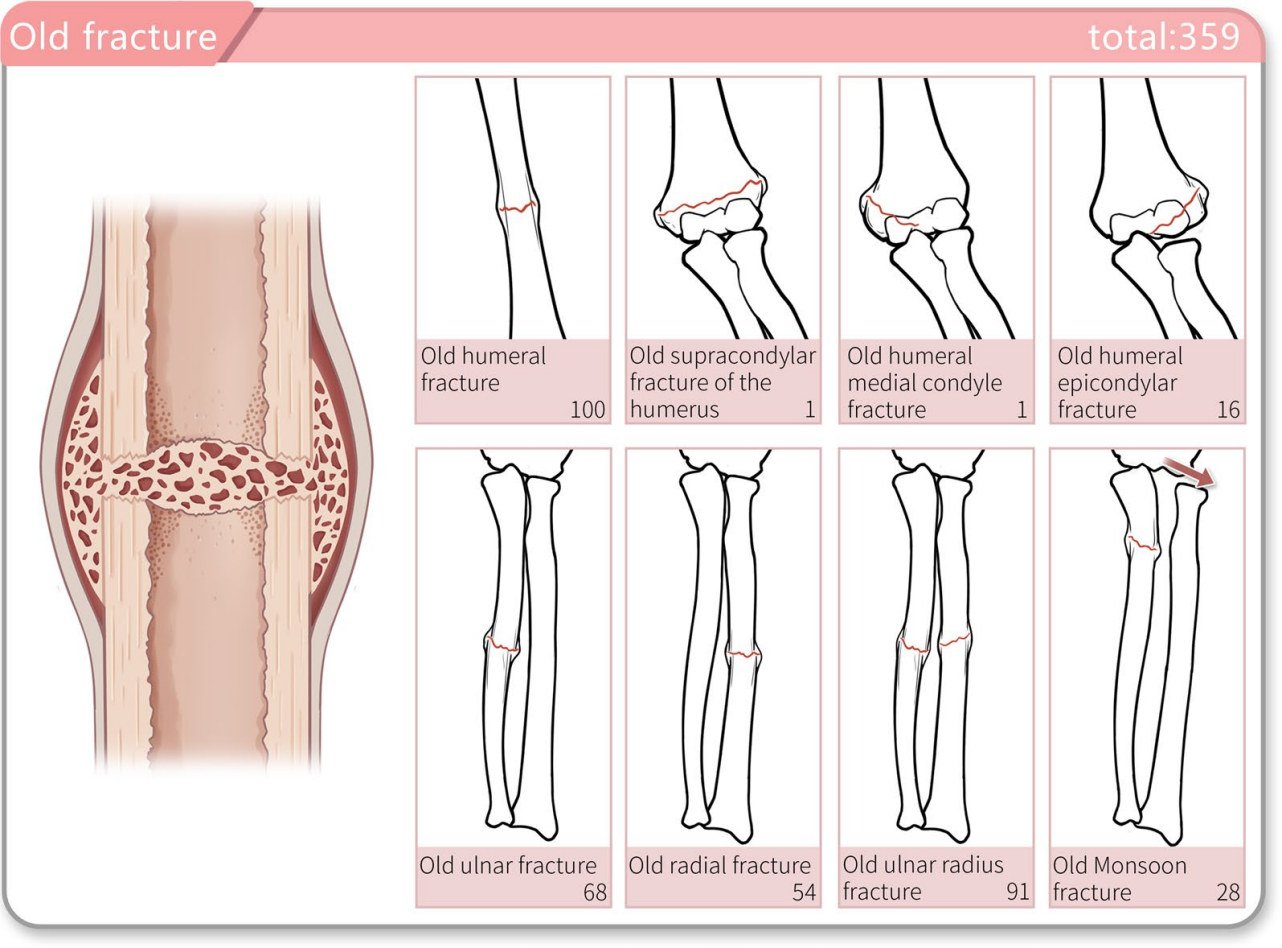
The old fracture sites of patients. This picture shows the common old fracture sites patients

Among all the 32,439 children, 359 presented with old fractures. A total of 118 were admitted to hospital with a diagnosis of old humerus fracture, of whom 86 were boys and 32 girls; the number of these cases was largest in preschool children, with 166 cases, and smallest in adolescents, with 4 cases. A total of 118 children were admitted with a diagnosis of old humerus fracture, including 100 with old humerus fracture, 16 with old lateral humeral condyle fracture, 1 with old humeral supracondylar fracture, and 1 with old medial humeral condyle fracture. In addition, 91 children were admitted with a diagnosis of old radial or ulnar shaft fracture, of whom 68 had old ulna fracture, 54 had old radius fracture, and 28 had old Monteggia fracture (Fig. 4).

Among the 32,080 children with fresh fractures, 262 had a coexisting diagnosis of joint dislocation, of whom 254 were affected by joint dislocation of the elbow, including 203 with elbow joint dislocation, 30 with humeroulnar joint dislocation, 10 with humeroradial joint dislocation, 10 with radioulnar joint dislocation, and 1 with a radial head dislocation. In addition, there were 4 children with shoulder joint dislocation, 2 with distal ulnar joint dislocation, 1 with a distal radial joint dislocation, and 1 with a wrist dislocation. Of the 203 children with elbow joint dislocations, 157 were boys and 46 were girls; 87 were school children, 74 were adolescents, and only 9 were infants. The most common fracture site for fractures with elbow joint dislocations was the distal humerus, with a total of 190 cases, and the most common distal humeral fractures with elbow dislocation were lateral humeral condyle (60 cases), medial humeral condyle (56 cases), and medial humeral epicondyle (43 cases).

### Causes of injuries

**Fig. 5:**
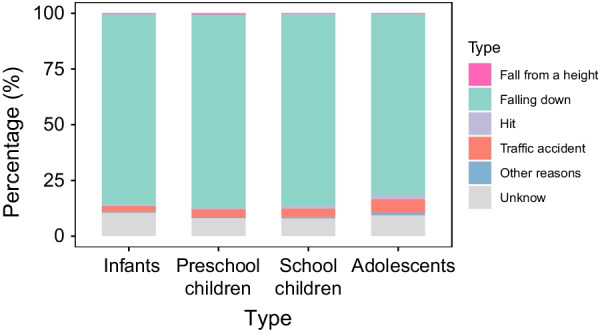
The epidemiology of age group according to different etiologies. This picture expresses the distribution characteristics of cause of injury among children of various age groups

For fresh fractures, the main causes were falling over, in 27,551 cases; traffic accidents, with 1176 cases; being hit or struck (290 cases); falling from a height (170 cases); and other reasons (194 cases), with unknown causes in 2699 cases. Falling over most commonly occurred in preschool children (12,312 cases—7653 boys and 4659 girls), followed by school children (8921 cases—6348 boys and 2573 girls), and was least frequent in the adolescent group (2273 cases, of whom 1993 were boys and 280 were girls) (Fig. 5).

Traffic accidents were another common cause of fractures, involving 1176 children with 779 boys and 397 girls. These cases were most commonly distributed in preschool children (505 cases), then in school children (386 cases), followed by adolescents (164 cases), and were least common in infants (121 cases). Admissions from traffic injuries peaked at 12h00, with a total of 405 cases. Fractures due to traffic accidents were more frequent in summer and autumn (721 cases).

### Treatment

Among the 32,080 children with fresh fractures, 806 did not undergo surgery. The remaining 31,274 underwent surgical treatment, of whom 29,028 had clear operative records and 2246 did not. Of the children without surgical treatment, 718 were treated with simple external plaster fixation, 80 with bandage fixation, and 8 with fracture site immobilization. Of the children with clear operative records, 10,962 received open reduction and 18,066 received closed reduction. In those with open reduction, Kirschner wire fixation was performed in 5829 cases, metal plate fixation in 415 cases, intramedullary nail fixation in 754 cases, screw fixation in 1425 cases, simple plaster fixation in 1145 cases, other internal fixations in 1338 cases, and external fixator fixation in 53 cases. In those with closed reduction, Kirschner wire fixation was performed in 10,050 cases, other internal fixations in 6117 cases, intramedullary nail fixation in 854 cases, screw fixation in 402 cases, simple external plaster fixation in 114 cases, metal plate fixation in 105 cases, traction in 93 cases, and external fixator fixation in 10 cases. The ratio of open reduction to closed reduction was 1:2.

All 359 children with old fractures underwent surgical treatment, with clear operative records available in 339. An open reduction was documented in 244 cases, and a closed reduction in 95 cases. In the open reduction group, 58 children had osteotomy; in addition, simple external plaster fixation was performed in 58 cases, other internal fixations in 93 cases, Kirschner wire fixation in 47 cases, screw fixation in 20 cases, metal plate fixation in 23 cases, external fixator fixation in 10 cases, and intramedullary nail fixation in 9 cases. In the closed reduction group, other internal fixations were performed in 32 cases, Kirschner wire fixation in 23 cases, screw fixation in 2 cases, and intramedullary nail fixation in 7 cases.

### Time and season of admission

**Fig. 6:**
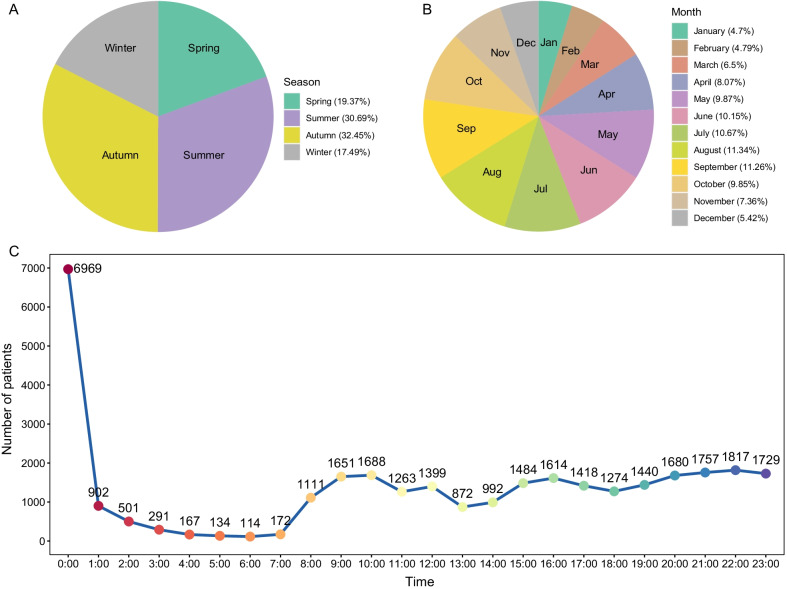
The exact hour, month, and season of the child's fracture. This picture illustrates the characteristics of the data distribution of admission of patients in **A** different season, **B** different months and **C** occurrence time of fractures

By analyzing the time of admission of all 32,439 children, we found that the number of children attending hospitals reached a peak of 6969 cases at 0 o’clock. Admissions peaked in autumn (August to October) with 10,527 cases, with the highest peak in August (3678 cases). The next highest number of admissions was in summer (May to July) with 9957 cases, peaking in July (3461 cases). Spring (February to April) saw 6282 cases, and 5673 cases occurred in winter (November to January) (Fig. 6).

### Geographic distribution

**Fig. 7:**
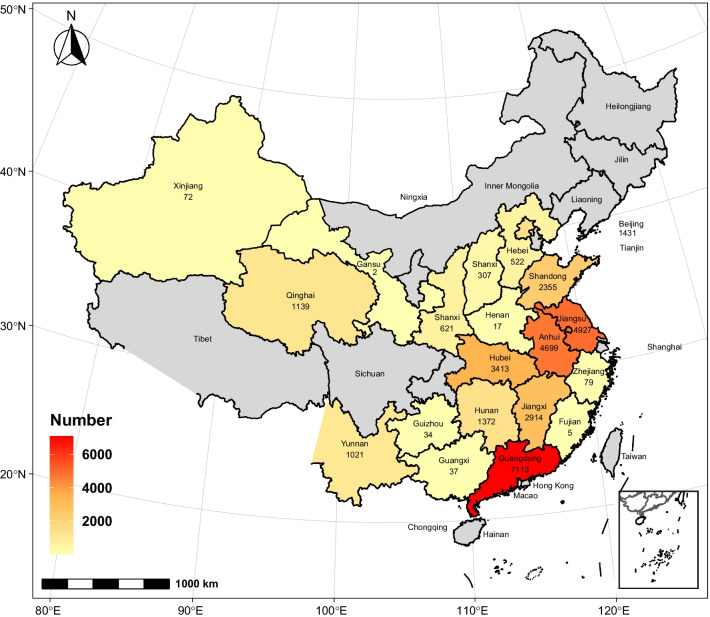
The geographical location of the hospital for 32,080 cases of freshly fractured children. This picture expresses the geographical hospital distribution of the number of fresh fractures in China

Among the 32,080 children with fresh fractures, 12,065 were distributed in East China (Anhui, Shandong, Jiangsu, Zhejiang, and Fujian provinces), 7716 in Central China (Hubei, Jiangxi, Hunan, and Henan provinces), 7150 in South China (Guangdong Province and Guangxi Zhuang Autonomous Region), 2260 in North China (Beijing, Hebei Province, and Shanxi Province), 1834 in Northwest China (Gansu Province, Qinghai Province, Xinjiang Uygur Autonomous Region, and Shaanxi Province), and 1055 in Southwest China (Guizhou and Yunnan provinces) (Fig. 7).

### Hospital costs and payment method

For the children with fresh fractures at all sites, the overall average total hospital cost was 10,994 yuan. For individual fracture sites, the highest average total hospital cost was 14,053 yuan for children with humeral shaft fractures, followed by 14,005 yuan for those with distal ulna fractures. The lowest average cost was 2021 yuan, for those with scapula fractures (Table 1). Significant differences were found in total hospital cost, nursing care cost, examination cost, surgery and anesthesia cost, treatment cost, medication cost, and materials cost between individual fracture sites (all *P* < 0.001; Table 2). For fresh fractures, material costs and surgery and anesthesia costs contributed most to total hospital costs.

**Table 1:** The composition of the median costs of hospitalization of inpatients with fresh fractures

Hospitalization cost (Yuan)	Clavicle		Scapula		Proximal humerus		Humeral shaft	
	Median IQR		Median IQR		Median IQR		Median IQR	
Nursing care	162.00	77.00	60.00	38.00	170.00	72.00	179.00	77.00
Examination	1225.70	878.00	1113.00	812.00	1416.00	1108.00	1494.10	1155.50
Treatment	658.90	364.00	434.35	250.25	1250.00	767.10	1178.20	709.85
Surgery and Anesthesia	2404.00	1895.00	0	0	2700.00	1432.00	2660.00	1447.00
Medication	1100.78	593.41	117.96	12.1	1274.53	636.74	1676.43	845.19
Material	3643.30	1030.28	164.71	98.40	5741.70	2281.50	5248.62	1494.33
Total hospital cost	11,075.90	7475.02	2021.13	1560.84	13,300.88	9947.55	14,052.67	9173.65

	Distal humerus		Proximal ulna		Ulnar shaft		Distal ulna	
	Median IQR		Median IQR		Median IQR		Median IQR	
Nursing care	173.00	77.00	77.00	45.00	136.00	61.00	110.50	61.88
Examination	1375.00	1114.50	1158.00	1012.00	1323.30	1018.00	1106.00	881.75
Treatment	1050.13	706.04	1613.20	860.00	996.90	683.04	1464.65	664.70
Surgery and Anesthesia	2924.00	1903.90	1903.90	1004.50	2350.00	1316.15	2371.00	1474.25
Medication	1118.13	660.24	328.29	140.57	1416.44	677.82	1276.02	601.67
Material	3729.88	1742.42	980.91	177.66	4831.25	1075.50	3181.48	774.24
Total hospital cost	10,919.47	8427.11	6907.42	5122.68	12,341.79	7550.66	14,004.87	8667.85

	Proximal radius		Radial shaft		Distal radius		Monteggia	
	Median IQR		Median IQR		Median IQR		Median IQR	
Nursing care	169.00	77.00	141.00	61.00	61.00	37.00	124.00	75.00
Examination	1492.00	1197.60	1370.00	1114.50	1128.00	996.00	1452.00	1113.50
Treatment	978.40	656.00	913.50	615.15	1784.00	867.45	1093.00	678.30
Surgery and Anesthesia	2174.00	1590.00	2266.00	1321.00	1306.00	900.00	2535.00	1306.00
Medication	1101.34	615.62	1229.93	736.99	197.01	99.97	1622.87	1002.20
Material	6100.00	4202.58	4801.65	1524.02	907.38	202.18	4255.38	1526.40
Total hospital cost	13,028.23	10,193.07	12,220.57	8342.33	6112.79	4722.72	12,153.23	7847.03

**Table 2:** Comparison of the economic factors of hospitalization for each fracture site of fresh fractures in hospitalized children

Fracture site	Proximal ulna	Ulnar shaft	Distal ulna	Humeral shaft	Proximal humerus	Distal humerus	Scapula
Total hospital cost	6907.42	12,341.79	14,004.87	14,052.67	13,300.88	10,919.47	2021.13
Nursing care	77	136	110.5	179	170	173	60
Examination	1158	1323.3	1106	1494.1	1416	1375	1113
Surgery and Anesthesia	1903.9	2350	2371	2660	2700	2924	0
Treatment	1613.2	996.9	1464.65	1178.2	1250	1050.13	434.35
Medication	328.29	1416.44	1276.02	1676.43	1274.53	1118.13	117.96
Material	980.91	4831.25	3181.48	5248.62	5741.7	3729.88	164.71

	Monteggia	Radial shaft	Proximal radius	Distal radius	Clavicle	Statistics	*P* value
Total hospital cost	12,153.23	12,220.57	13,028.23	6112.79	11,075.9	1340.394	0.00
Nursing care	124	141	169	61	162	902.724	0.00
Examination	1452	1370	1492	1128	1225.7	453.457	0.00
Surgery and Anesthesia	2535	2266	2174	1306	2404	1023.072	0.00
Treatment	1093	913.5	978.4	1784	658.9	600.698	0.00
Medication	1622.87	1229.93	1101.34	197.01	1100.78	1031.104	0.00
Material	4255.38	4801.65	6100	907.38	3643.3	1449.819	0.00

For the 359 children with old fractures, the overall average total hospital cost was 15,151 yuan. The highest average total hospital cost was 20,698 yuan for children with old ulna fractures, followed by 15,686 yuan for those with old Monteggia fractures. The lowest average cost for management of old fractures was 10,939 yuan for old radius fractures (Table 3). Significant differences were observed in total hospital cost, nursing care cost, examination cost, surgery and anesthesia costs, treatment cost, medicine cost, and material costs between old fracture sites (all *P* < 0.001; Table 4). For old fractures, the major factors affecting total hospital cost remained cost of materials and surgery/anesthesia costs.

**Table 3:** Comparison of the economic factors of hospitalization of old fractures in hospitalized children

Fracture site	Old ulna fracture	Old radial or ulnar shaft fracture	Old humerus fracture	Old Monteggia fracture	Old radius fracture	Statistics	*P* value
Total hospital cost	20,697.65	13,192.15	13,622.49	15,686.03	10,939.36	66.16	0.00
Nursing care	265.50	93.00	172.00	299.00	95.50	118.52	0.00
Examination	1669.85	1337.60	1626.00	1436.00	1202.50	68.04	0.00
Surgery and Anesthesia	3667.45	2110.00	3637.00	3543.50	2012.50	148.05	0.00
Treatment	1216.85	1059.30	1136.75	765.55	1225.22	24.73	0.00
Medication	1254.72	1039.45	932.90	2172.77	1110.08	65.85	0.00
Material	10,451.00	4458.37	5000.70	7578.10	4126.01	49.17	0.00

**Table 4:** The composition of the median costs of hospitalization of inpatients with old fractures

Hospitalization cost (Yuan)	Old ulna fracture		Old radial or ulnar shaft fracture		Old humerus fracture		Old radius fracture		Old Monteggia fracture	
	Median	IQR	Median	IQR	Median	IQR	Median	IQR	Median	IQR
Nursing care	265.50	111.50	93.00	60.00	299.00	138.25	172.00	93.00	95.50	48.75
Examination	1669.85	1249.30	1337.60	1072.00	1436.00	1212.00	1626.00	1287.60	1202.50	1077.00
Treatment	1216.85	885.53	1059.30	786.57	765.55	534.53	1136.75	838.40	1225.22	725.31
Surgery and Anesthesia	3667.45	2924.00	2110.00	1232.00	3543.50	2606.25	3637.00	2965.00	2012.50	1146.00
Medication	1254.72	322.96	1029.45	472.33	2172.77	1308.55	932.90	317.78	1110.08	222.88
Material	10,451.00	5409.19	4458.37	748.36	7578.10	5428.81	5000.70	2963.97	4126.01	901.59
Total hospital cost	20,697.65	14,011.56	13,192.15	5493.94	15,686.03	12,566.27	13,622.49	11,374.40	10,939.36	7064.85

In comparison of all hospital costs between the 32,080 children with fresh fractures and the 359 children with old fractures, significant differences were observed for total hospital cost (*P* < 0.001), as well as nursing care cost, examination cost, surgery and anesthesia costs, medication cost, and cost of materials (all *P* < 0.001), but not in treatment cost (*P* = 0.702).

**Fig. 8:**
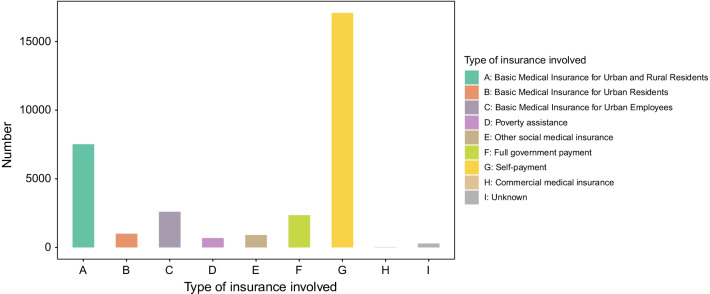
The proportion of different type of insurance in all patients. This picture shows the proportion of different type of insurance in all patients

Fresh and old Monteggia fractures showed significant differences in total hospital cost, nursing care cost, surgery and anesthesia costs, treatment cost, and cost of materials (all *P* < 0.001). Fresh and old ulna fractures showed significant differences in total hospital cost, nursing care cost, examination cost, surgery and anesthesia costs, medication cost, and cost of materials (all *P* < 0.001). Fresh and old radius fractures showed only a significant difference in material costs (*P* < 0.001). Fresh and old humerus fractures showed significant differences in total hospital cost, examination cost, surgery and anesthesia costs, medicine cost, and cost of materials (all *P* < 0.001). Among the 32,439 children with fresh and old fractures, full self-payment accounted for the highest proportion of all payment methods, with a total of 17,088 cases and an average cost of 11,111 yuan (Fig. 8).

## Discussion

In this study, we made a number of observations, described as follows. For both fresh and old upper limb fractures, more boys than girls were hospitalized in all age groups, and the number of hospitalized children was largest in the preschool (2–6 years) group. The most common fresh fracture site was the distal humerus, especially the supracondylar area of the humerus, and the most common cause of fresh fractures was falling over. Children with fresh elbow fractures were mostly aged between 2 and 6 years (preschool group). Elbow joint dislocations were the most common joint dislocation type accompanying fresh upper limb fractures and typically occurred with distal humerus fractures. Fresh fractures were mainly treated with closed surgical reduction, with a ratio of two cases with closed reduction to one case with open reduction, and old fractures were mainly treated with surgical open reduction, 16.16% of whom had osteotomy. Fresh fracture cases were most commonly distributed in the East China region, and admissions from fresh and old fractures were more frequent in autumn and summer. Admissions peaked at 0 o’clock. Significant differences in total hospital cost, nursing care cost, examination cost, surgery and anesthesia costs, treatment cost, medication cost, and cost of materials were found between hospitalized children with fresh fractures and those with old fractures; for both fresh and old fractures, cost of materials was the principal factor affecting total hospital cost, followed by surgery and anesthesia costs. Average total hospital costs of fresh fractures (highest for humeral shaft fractures) were 25% lower than the average for old fractures (highest for old ulna fractures). Among all children, full self-payment, at 53% of children, accounted for the highest proportion of all payment methods.

### Age and sex

Fracture incidence peak in children varies across countries. Fracture incidence in American children peaks at the age of 10–14 years [10]. An Israeli study reported that the peak age of fractures was 10–11 years in girls and 12–13 years in boys, and incidence in boys was higher than girls in all age groups [11]. A Chinese study of upper limb fractures in children observed a peak incidence between the ages of 12 and 18 years in boys and 6 and 12 years in girls [8]. Another Chinese study showed fracture incidence as peaking between 6 and 12 years in both boys and girls [12]. However, a study by Cheng et al. [13] reported a peak age of fractures of about 3 years in Chinese children. Our research also showed a peak age of 3–6 years for both sexes, and incidence was higher in boys than girls in all age groups.

A British study found that the incidence of fractures increased with age in both boys and girls but decreased after the age of 11 years in girls [14]. Consistent with this finding, our data showed that the number of girls with fractures decreased after the age of 11 years, with the ratio of boys to girls as high as 6.7:1 in this age group. Boys may be more active and curious by nature and show a tendency to higher-risk outdoor activities; this may make them more prone to upper limb fractures than girls in all age groups, despite greater musculoskeletal strength in boys. Therefore, to prevent fractures in boys, safety education for parents and guardians should be strengthened, especially for those with children aged 3–6 years.

### Fracture site

One study has reported that forearm fractures are the most common fracture location in children, especially ulnar and radial shaft fractures [15]. Other studies have indicated that the major fracture site varies in children according to ages, with the most common fracture under the age of 2 years being distal radius fracture, humeral supracondylar fracture between 3 and 6 years, distal radius fracture from 7–12 years, and phalanx fractures between 13 and 17 years [16]. However, an epidemiological survey of elbow fractures in Iranian children suggested that the most common fracture site was the supracondylar area of the humerus [17]. Distal humerus fractures have also been reported the most common upper limb fracture type in adolescents, accounting for approximately 60% of all elbow fractures [18]. Our study similarly showed that distal humerus fractures were the most common fracture across all age groups, with the highest percentage being humeral supracondylar fractures. The humeral supracondylar area is located at the junction of cancellous and cortical bone, and there is an anteversion angle of 30° between the humeral shaft axis and the humeral condyle axis. During a fall, stress tends to be concentrated on the supracondylar area, leading to high risk of humeral supracondylar fracture.

Elbow fractures are reported to account for 40–48% of upper limb fractures [19]. A recent Indian study demonstrated elbow fractures as the most common fracture type in children [20], in line with a Japanese study [21]. Consistently, elbow fractures represented 61% of all upper limb fractures in our study population. Elbow fractures are rarely life threatening but can cause serious complications. For example, supracondylar fractures can lead to nerve and vessel injuries, cubitus varus deformity, and Volkmann contracture; lateral humeral condyle fractures may result in bone nonunion, valgus deformity, and tardy ulnar nerve palsy.

In children, the skeleton has yet to mature, the epiphyseal plate has yet to close, and the bone has yet to fully harden, so the bone has less strength to resist external force than soft tissues surrounding the elbow joint such as ligaments. When the elbow sustains severe trauma, an elbow fracture develops easily, whereas the incidence of elbow dislocation, which is generally caused by high-energy trauma but is a relatively rare condition in clinical practice, is low [22, 23]. In our study, 254 children suffered fractures with joint dislocations of the elbow, including 203 with elbow joint dislocation, 30 with humeroulnar joint dislocation, 10 with humeroradial joint dislocation, 10 with radioulnar joint dislocation, and 1 with a radial head dislocation. Falling over was the major cause of elbow dislocation, with a total of 206 cases and was the cause in 81% of cases. Some studies have reported that most childhood elbow dislocations are accompanied by intra-articular fractures [24, 25]. In our study, the most common fracture site with elbow dislocation was the distal humerus, and the highest percentage was intra-articular fractures. On radiographs, the dislocated elbow joint can be obscured by the normal bone and, in addition, the presence of six epiphyses (in the humeral lateral condyle, trochlea, lateral epicondyle, medial epicondyle, radial head, and ulnar olecranon) increases the possibility of a misdiagnosis or missed diagnosis in children with fractures accompanied by elbow joint dislocation [26].

### Causes of injuries

Although childhood fractures have a variety of causes, most fractures result from falling over during play or sport. Falling has been cited as the major cause of childhood fractures in other studies, including one from Scotland [15, 27]. Our study also demonstrated falling over as the major cause of upper limb fractures in children, especially among those aged between 3 and 6 years. With increasing locomotor ability at this age, children often fall during play and habitually stretch out their upper limbs to protect themselves, leading to the occurrence of upper limb fractures. Fall-induced childhood fractures commonly occurred at home (53%), school (16.5%), and in amusement parks (10%) [28]. Close attention should be paid to children in these places to prevent falls or other accidental injuries and reduce the occurrence of upper limb fractures.

Traffic accidents are another common cause of injury. A Malaysian study reported that traffic accidents were the second leading cause of injuries (30.7%) among children hospitalized with trauma [29]. An investigation of injury mortality involving 1728 children under 14 years old in Britain showed that traffic accidents were the leading cause of mortality, responsible for more than 40% of the deaths [30]. Canadian researchers collected data on 237 children with severe trauma from 1996 to 2000 and found that the most common cause was traffic accidents (39%) [31]. In our study, traffic accident was the second highest cause of fracture and was most common in those aged between 3 and 6 years old. Traffic injuries occurred mainly in summer and autumn, which could be due to the increased outdoor activity of children in these two seasons. In addition, the peak time of admission for traffic injury was 12:00. This may be because drivers are fatigued after working during the morning, resulting in reduced safety awareness. In addition, traffic officers rest at noon, and so traffic management may be relatively lax at this time. Guardianship of children during outdoor activities should be strengthened, and traffic officer numbers increased to maintain traffic order.

### Time and season of admission

Childhood fractures have significant seasonal patterns [12]. Fracture incidence in children peaks in spring and summer for all fracture sites [32], and most studies describe an increase in the incidence of childhood fractures in spring and summer [33, 34]. However, some studies reported the peak of pediatric fractures in autumn and winter [35, 36]. Swedish research observed a decline in fracture incidence in July, and based on the observation that play was the predominant activity at fall injury, the researchers reasoned that children do not continue regular daily activities during school holidays, and their decreased activity levels could be a contributing factor to declined fracture incidence at this time [3]. In our study, the number of children with upper limb fractures was largest in summer and autumn, with a significant reduction in winter. This may be because suitable weather and long daylight hours in summer and autumn promote outdoor activities in children, and the thin clothing worn in warmer weather has no collision energy-absorbing buffer capacity in the case of accidental injuries. Although Chinese children have the longest holidays (summer vacation, National Day holiday, etc.) in summer and autumn, we observed no decline in the number of fractures, as opposed to the Swedish research. In fact, Chinese children have more time for outdoor activities during vacations than in school, thus increasing the risk of unintentional injuries and fractures. Contrary to our results, a Danish study described a peak incidence of pediatric upper limb fractures in winter [32], which may be attributed to the sports of sledding, skating, and skiing. In China, these sports are not widespread among young children, and the average winter temperature in some regions is not low enough provide an environment for these activities.

### Hospital costs

Childhood unintentional injuries are a major global public health problem, increasing disability and mortality and imposing a huge economic burden [37]. The direct and indirect costs of childhood unintentional injuries can be categorized as medical care, hospital admission time, medical insurance, vehicle maintenance, legal costs, school absence, and parental income loss owing to childcare. In the USA, the hospital cost of childhood unintentional injuries reached 9,550,700 US dollars in 2003 [38]. Chinese researchers reported that the total hospital cost for accidental injuries in children from 2002 to 2003 was 1,033,876 US dollars, with an average cost of 166 US dollars per child. Hospital cost for fractures was the largest component at 306,572 US dollars [39]. A study in Norway revealed that children under 14 years old accounted for 31% of all accidental injury cases, with an average cost of 1856 US dollars per child [40]. In our study, the average total hospital cost was 10,994 yuan for fresh fractures and 15,151 yuan for old fractures.

Cost factor analysis in our study showed that material costs and surgery and anesthesia costs were the key factors influencing total hospital costs. Old fractures were treated mainly with surgical open reduction, 16.16% of whom had osteotomy which incurred higher hospital costs, thus causing a heavy economic burden for most families. We screened for children with old upper limb fractures by ICD code, so we were unable to ascertain the cause of fracture in these children, which is a limitation of our study. Hand phalanx (22.0–26.4%) and elbow (11.4–15.3%) are the most common sites for misdiagnosis and missed diagnosis in children with fractures [26, 41], and 80% of these have been found to be because of emergency doctors’ errors in reading radiographs [42]. In our child population, the distal humerus was the most common fracture site. Because the anatomical structure of the elbow and surrounding structures is more complex in children than in adults, emergency doctors may misdiagnose or miss a fracture when reading children’s upper limb radiographs, delaying the optimal timing of treatment for fresh fractures. Diagnostic training for emergency doctors on pediatric fractures could be strengthened. Doctors should be encouraged to perform routine radiographic examination of the contralateral asymptomatic elbow for comparison when diagnosis is unclear, or order an MRI or CT scan. In addition, there are some differences in the treatment of pediatric fractures and adult fractures, and incorrect treatment that disregards the plasticity of pediatric fractures in relation to growth will affect fracture healing in children. More provision of and better access to specialized pediatric hospitals or pediatric trauma centers in general hospitals would provide children with more timely and effective care, avoid unhealed or poorly managed fractures, and reduce economic costs for their families.

### Medical insurance

The medical security of children has long received universal attention across the world. In the USA, approximately 89% of children under 18 years old are covered by medical insurance. In 1997, the US Congress enacted federal government funding to state governments to establish insurance programs for children without any extra cost for low-income families [43]. In a 2003 US survey of 1475 children with unintentional injuries, only 8% of families paid medical expenses themselves [38]. In Japan, all citizens benefit from universal health insurance and are free to choose medical institutions [44]. At present, China has established an institutional framework for universal medical insurance. The basic medical insurance has covered 1.345 billion people in 2018, which is 95% of the total population. However, the level of protection is still relatively limited. Affected by the dual system of urban and rural areas, the difference between urban and rural children's medical insurance has always existed in my country, such as the low level of child protection, the shortage of drugs, and the shortage of pediatricians [45].

At this stage, medical security, as an important component of various social security systems in my country, has played an important role in solving the medical and health problems of our people. However, due to the particularity of the child group, the limited number of participants and the level of protection, the lack of some system functions, coupled with the large regional differences in implementation, and the lack of unified information management, the medical security treatment for children is uneven. In addition, some urban residents and employees have their children’s medical costs reimbursed by insurance provided by parents’ work institutions or directly by the government; however, achieving comprehensive insurance is difficult because of narrow cover and low standards. Commercial insurance for children is profit-oriented and driven by the principle of “more fee for more sum insured, but no fee for no sum insured,” and offers relatively narrow cover and few options for child trauma. Medical security is even worse in migrant children. Previously, more than one-third of American children received public health insurance through the Medicaid and the Children's Health Insurance Program (CHIP); however, undocumented immigrant children did not receive public health insurance. In recent years, California has expanded children's access to public medical insurance. Regardless of their immigration status, as long as they meet the eligibility, for insurance, they can participate in the CHIP plan and reduce the financial burden of medical treatment [46]. American makes great efforts to secure children's health insurance, and the CHIP program is a major success. This success is reflected in high rates of coverage and access to health care among children, including those from low-income families [47]. In our study, the treatment of 17,088 children (52.7%) was funded by full self-payment, with an average hospital cost of 11,111 yuan. According to data released by China’s National Bureau of Statistics in the first half of 2021, the national median per capita disposable income was 14,897 yuan. Thus, hospital costs have a significant economic impact on families who must make full self-payment and can lead to delayed care in poorer families and result in unhealed fracture or malunion in some children. The system not only hinders the timely treatment of children with fractures, but also increases the cost of treatment. China must learn from international experience and create a government-led comprehensive medical security system for children or make adjustments to the existing general medical security system, integrating social resources to meet the diverse needs of medical security for children. The capacity of pediatric services must be strengthened in primary medical institutions, fundamentally shoring up the weak links in children’s medical security and providing better services for children. Simultaneously, central government should provide financial incentives to local governments to promote the health care of migrant children. Local governments could encourage the participation of migrant children in the urban medical insurance system by lowering premiums to reduce their economic burden from medical care.

## Conclusion

Upper limb fractures in children are an important public health problem faced by countries across the world. To prevent upper limb fractures in children, authorities need to strengthen safety education for children, parents, communities, and schools; enhance protective measures in children’s activities; and perfect relevant legislation, based on the distribution characteristics of age, sex, and cause of injury. In our study, for fresh and old fractures, material costs and surgery and anesthesia costs contributed most to total hospital costs. The overall average total hospital cost is higher in children with old fractures than in children with fresh fractures. Among all children, full self-payment, at 53% of children, accounted for the highest proportion of all payment methods. Hospital costs are a headache for those families who will pay on their own. It can lead to a delayed treatment and unhealed fractures or malunion in some children. To provide better medical services for children, authorities must improve the allocation of health resources, establish a comprehensive medical security system for children, and set up more child trauma centers. Doctors require standardized training on child trauma management. This would avoid misdiagnosis or missed diagnosis of upper limb fractures and incorrect treatment that disregards the plasticity related to growth that delays the optimal timing of treatment and results in unhealed or malunion fractures. These combined measures will improve children’s quality of life and reduce the public health burden.

## Data Availability

The datasets generated and/or analyzed during the current study are not publicly available due to limitations of ethical approval involving the patient data and anonymity but are available from the corresponding author on reasonable request.
